# Does the appearance of the *Magenstrasse* depend on the amount of water consumed?

**DOI:** 10.1016/j.ijpx.2025.100365

**Published:** 2025-07-23

**Authors:** Linus Großmann, Johanna Cyrus, Stefan Senekowitsch, Toni Wildgrube, Theodora Tzakri, Marie-Luise Kromrey, Werner Weitschies, Michael Grimm

**Affiliations:** a University of Greifswald, Institute of Pharmacy, Department of Biopharmaceutics and Pharmaceutical Technology, Felix-Hausdorff-Str. 3, 17489 Greifswald, Germany; b University Hospital Carl Gustav Carus, Institute for Diagnostic and Interventional Radiology, Fetscherstrasse 74, 01307 Dresden, Germany

**Keywords:** *Magenstrasse*, Stomach road, MRI, Saliva, Food effect, Gastric emptying, Water, Tablet, Capsule

## Abstract

The *Magenstrasse* (stomach road) is a phenomenon describing the rapid evacuation of water drunken after a solid meal from the stomach. So far, its existence has been demonstrated for water volumes of 150 mL or more. The aim of this three-arm, randomised, cross-over, 12-subject study was to investigate whether the *Magenstrasse* is also present for smaller water volumes. For this purpose, gastric emptying of 50, 100 or 150 mL of water that was administered after a light meal was determined using MR imaging. With each dose of water, a fast-dissolving compression coated tablet containing caffeine and iron oxide as well as a hard capsule containing stable isotope labelled caffeine and medium-chain triglycerides were administered. This made it possible to determine the initial localization of the respective forms in the stomach on MR images as a function of the amount of water drunk, and also to determine the emptying rates of the two caffeine variants using saliva samples that were obtained in the study and quantified using LC-MS/MS. Gastric emptying of the ingested water was rapid and usually completed after approximately 20 min, regardless of the applied volume. In contrast to the consumed water, gastric emptying of natural caffeine and stable isotope labelled caffeine was delayed. The capsule usually floated on liquid and chyme, whereas the compression coated tablet was often embedded in chyme.

## Graphical abstract

**Figure:**
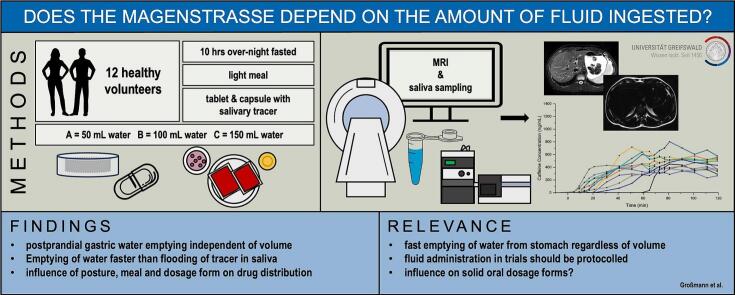

## 1 Introduction

The *Magenstrasse* or stomach road describes the phenomenon of rapid emptying of water from the stomach in the postprandial state (Grimm et al., 2017). This emptying occurs mainly along the entire stomach wall or the *Plicae Gastricae* of the lesser curvature towards the antrum, pylorus and finally into the duodenum as studies in humans and dogs have shown (Koziolek et al., 2014; Jefferson, 1915; Scheunert, 1912; Malagelada et al., 1979; Malagelada, 1977; Almutairi et al., 2023; Kiyota et al., 2022a; Koziolek et al., 2016). Another mechanism involved could be an adaptive relaxation of the stomach wall with increasing wall tension and hydrostatic pressure of the applied fluid volume (Grimm et al., 2017). Due to the positioning of orally applied dosage forms in the stomach and their resulting contact time with dissolution or liberation media, the *Magenstrasse* is thought to have a significant influence on the variability of drug absorption in the postprandial state and may therefore influence plasma levels (Koziolek et al., 2016; Weitschies et al., 2005; Weitschies et al., 2008). For bioavailability and bioequivalence studies under fasting and fed conditions FDA (US Food & Drug Administration) guidances recommend drug administration together with 240 mL of water, EMA (European Medicines Agency) advices at least 150 mL of water (FDA, 2002; EMA, 2010; FDA, 2003).

However, real-life dosing conditions often differ from the amounts prescribed in the guidelines. Hens et al. describe for a Dutch population that most people take their medication with only half a glass of water (100–120 mL). In fact, the second most reported intake volume was only a sip of water (Hens et al., 2017). This is supported with a recent study by Sarwinska et al. for a northern German population of elderly people. Most subjects took their medication with 100–200 mL of water and 21 % with just a few sips (about 50 mL) (Sarwinska et al., 2024).

It has been shown that the gastric emptying rates of 20, 50 and 240 mL of water consumed under fasting conditions are well comparable (Grimm et al., 2023a; Grimm et al., 2023b). Based on the assumed mechanisms underlying the stomach road, the question arises as to whether there is a lower volume limit for the development of the stomach road. The aim of the present study was therefore to investigate whether the *Magenstrasse* also occurs when drinking volumes of water of 50, 100 and 150 mL. In an MRI (magnetic resonance imaging) study in healthy volunteers, the stomach volume was to be determined after eating a test meal and drinking 50 mL, 100 mL and 150 mL of water. Furthermore, by using caffeine as a saliva marker, the influence of the drinking volume and the site of disintegration in the stomach of rapidly disintegrating dosage forms taken with water was to be investigated. The dosage forms used were gelatine hard capsules filled with 25 mg ^13^C_3_-caffeine and compression-coated tablets containing 25 mg regular caffeine (Tzakri et al., 2023). Both dosage forms included a compartment with an MR identifiable marker substance to locate them in the stomach.

## 2 Materials & methods

### 2.1 Compression-coated tablets

Compression-coated tablets were manufactured as described elsewhere (Tzakri et al., 2023). Briefly, the inner core contains regular caffeine and black iron oxide and the outer coating acts as a rapidly disintegrating protective layer for the core. This is intended to prevent caffeine contamination of the oral cavity during the swallowing process. The iron oxide in the core was used for visualisation in the MR images by means of the characteristic susceptibility artefact that is visible in the used MRI sequences. The components of the tablet are given in Table 1. The tablets were biconvex-shaped and had a diameter of 9 mm and a hight of 5 mm. Tablet hardness, mass uniformity and in vitro disintegration and dissolution tests were in line with the results of Tzakri et al. (Tzakri et al., 2023). The compression-coated tablet had an approximate density of 1.18 g/mL.

**Table 1:** Composition of the compression-coated tablets.

Excipient	Proportion in the formulation [%]	Supplier
**Core**		
Caffeine	49 (25 mg per tablet)	Caesar & Loretz GmbH, Germany
Saccharine sodium	20	Caesar & Loretz GmbH, Germany
Avicel® PH 102	21	F. Hoffmann-La Roche AG, Switzerland
Vivasol®	5	JRS Pharma GmbH & Co. KG, Germany
Aerosil®	2	F. Hoffmann-La Roche AG, Switzerland
Magnesium stearate	1	F. Hoffmann-La Roche AG, Switzerland
Black iron oxide	2	Caesar & Loretz GmbH, Germany
**Coat layer**		
Methocel E4M®	2	Caesar & Loretz GmbH, Germany
Magnesium stearate	1	F. Hoffmann-La Roche AG, Switzerland
Prosolv® SMCC HD 90	97	JRS Pharma GmbH & Co. KG, Germany

### 2.2 Gelatine capsules

Size 0 gelatine hard capsules (WEPA Apothekenbedarf GmbH & Co KG, Germany) were each filled manually with 25 mg of ^13^C_3_-caffeine (Sigma-Aldrich Chemie GmbH, Germany) using an analytical balance (*CPA1003S,* Sartorius AG, Germany). A size 3 gelatine capsule (WEPA Apothekenbedarf GmbH & Co KG, Germany) was filled with medium-chain triglycerides (MCT) (Caesar & Loretz GmbH, Germany) to the upper edge of the lower half of the capsule (about 240 mg) and was also placed in the size 0 gelatine capsule to enable the capsule to be located in the stomach in the employed MRI sequences. The capsule had an approximate density of 0.68 g/mL.

### 2.3 Study protocol

A randomised, three-arm, cross-over study with 12 healthy subjects (5 female, 7 male, 26.1 ± 5.1 years, 74.3 ± 11.8 kg, 179.9 ± 7.0 cm, 22.9 ± 3.0 kg/m^2^) was conducted at the Department of Diagnostic Radiology and Neuroradiology, University Medicine Greifswald (Germany). The study was approved by the Ethics Committee of the University Medicine Greifswald under the registration number BB 080/23 and registered in the German Clinical Trials Register under number DRKS00033695. The study protocol conformed to the Declaration of Helsinki in its most recent form (Fortaleza October 2013). Randomisation was partially counterbalanced in blocks of three using a random number generator for study arms A - C to ensure even distribution.

In study arm A, 50 mL, in study arm B, 100 mL, and in study arm C, 150 mL of tap water was consumed 30 min after intake of a test meal in an upright position. In each study arm, one of the hard capsules and one of the compression-coated tablets were taken simultaneously with the respective amount of water. After 60 min additional 240 mL water were administered in an upright position in all study arms. All subjects attended the study day after an overnight fast (at least 10 h) and a caffeine fasting period of 72 h.

### 2.4 Meal

A standardised light meal (596 kcal) was served. It consisted of two slices of toasted wheat bread (Sammy's Super Sandwich, HARRY-BROT GmbH, Germany), spread with 10 g of butter (Meggle Alpenbutter, MEGGLE GmbH & Co. KG, Germany) and 15 g of strawberry jam (Erdbeerkonfitüre extra, Gut & Günstig, EDEKA Group, Germany) on each slice and 250 g of low-fat strawberry yoghurt (Fettarmer Joghurt 1,5 % Erdbeere, Gut & Günstig, EDEKA Group, Germany) as well as 120 mL of orange juice (Orangensaft, 100 % Fruchtgehalt, Gut & Günstig, EDEKA Group, Germany). The consumption of the test meal had to be finished within 15 min.

### 2.5 MRI

Magnetic resonance imaging was performed with a 3 Tesla Siemens MAGNETOM Vida MRI, an 18-channel body coil and 32-channel spine coil (Siemens Healthineers AG, Germany). Measurements were performed in a head-first supine position over the entire period of 60 min after ingestion of the water (*t* = −6, 4, 8, 12, 16, 20, 30, 40, 50, 58 min) with no getting up in between the measurements. A localizer sequence was used to position the field of view (FoV) in the stomach region. A transversal T2-weighted HASTE (Half Fourier-Acquisition Single-Shot Turbo Spin Echo) sequence was used to determine the gastric content volume and to visualize the susceptibility artefact from the tablet. Additionally, a transversal T1-weighted Dixon VIBE (Volume-Interpolated-Breath-Hold-Examination) sequence was used to locate the capsule from the resulting fat signal of the MCT. All sequence parameters are given in Table 2.

**Table 2:** Sequence parameters for MR image acquisition.

Sequence parameter	HASTE	Dixon VIBE
Repetition time [ms]	800	7.2
Echo time [ms]	86	1.3
Flip angle [°]	139	30
Slice thickness [mm]	5	4
Matrix [pixel]	384 × 228	704 × 396
Field of View [mm]	500 × 296	550 × 309
Voxel size [mm^3^]	8.45	2.43

Stomach volumes were acquired using Horos DICOM software (version 3.3.6) by encircling the outer contour of the stomach content without possible air compartments in every slice. Addition of the marked area (ROI – Region of Interest) of every slice with known voxel dimensions lead to a stomach content volume (GCV) for every measuring time point.

### 2.6 Salivary tracer & LC-MS/MS

Saliva samples were acquired immediately after the MRI measurements at the time points t = −5, 5, 9, 13, 17, 21, 31, 41, 51, 59, (at *t* = 60 min 240 mL water were ingested) 65, 69, 73, 77, 81, 91, 101, 111 and 119 min. Saliva samples were collected by drooling into 2 mL Eppendorf Tubes (Eppendorf GmbH, Germany) under supervision of the study personnel and immediately frozen at −80 °C until analysis. For LC-MS/MS analysis, samples were thawed under room temperature for one hour. They were then vortexed for 30 s at 4000 rpm and centrifuged (Biofuge® pico, Heraeus, Hanau, Germany) at 13000 rpm for 10 min. In the next step, 100 μL of the supernatant was transferred into a new 1.5 mL micro tube (Eppendorf GmbH, Germany) and then spiked with 25 μL of the internal standard (d9-caffeine, 4.0 μg/mL in water) and vortexed for a further 30 s at 4000 rpm. The proteins contained in the saliva samples were precipitated by adding 200 μL of a mixture of acetonitrile (Fisher Scientific, Loughborough, UK) and 1 % formic acid (Sigma-Aldrich Chemie GmbH, Steinheim, Germany). Subsequently, the mixture was vortexed again for 30 s at 4000 rpm, cooled for five minutes at −20 °C and finally centrifuged for 5 min at 13000 rpm. From the resulting precipitated saliva, 200 μL of the supernatant was again removed and transferred to a 1.5 mL HPLC vial (VWR, Radnor, USA) containing 300 μL of a mixture of deionised water and 1 % formic acid. The stock solutions for calibration and quality control were prepared in water. The quality controls were prepared after 1:20 dilution with analyser-free blank saliva.

10 μL of the processed samples were injected to a LC-MS/MS system (Shimadzu Corporation, Kyoto, Japan). The LC-MS/MS system consisted of a LC-40B X3 solvent delivery module, a SIL-40C X3 auto sampler, a CTO-40S column oven, a SPD-40 UV–Vis detector, a FCV-20AH_2_ valve unit and a LCMS-8060 mass spectrometer equipped with an ESI ionization unit.

The LC-system was equipped with a Phenomenex Kinetex® 2.6 μm PS C18 100 Å 150 × 2.1 mm column (Phenomenex, Torrance, USA) protected by a SecurityGuard™ ULTRA Cartridge (Phenomenex, Torrance, USA) that was connected to a SecurityGuard™ ULTRA Holder (Phenomenex, Torrance, USA). Water (Fisher Scientific, Loughborough, UK) containing 0.1 % formic acid (VWR, Radnor, USA) and methanol (Fisher Scientific, Loughborough, UK) (B) were used as eluents for the mobile phase at a flow rate of 0.4 mL/min and isocratic conditions (22 % B). The oven temperature was set to 40 °C. The total run time of the method was 4.5 min. Using the high-pressure switching valve, only the eluate from 1.3 to 4.5 min was directed to the mass spectrometer.

The detection of the analytes was performed in the positive multiple reaction monitoring (MRM) mode. The MRM transitions used for quantification of the analytes, the corresponding collision energies (CE) and the retention times are summarized in Table 3. Furthermore, the following parameters were applied: nebulizing gas flow 3 L/min, heating gas flow 10 L/min, drying gas flow 3 L/min, interface temperature 300 °C, desolvation temperature 526 °C, desolvation line temperature 250 °C and heat block temperature 400 °C. The interface voltage of the ESI source was 4.0 kV.

**Table 3:** Analytes detection with positive multiple reaction monitoring.

Analyte	MRM-transition	Collision energy	Retention time
^13^C_3_-Caffeine	198.20 *m*/*z* ➔ 140.10 m/z	- 20.0 eV	3.30 min
Caffeine	194.90 m/z ➔ 138.05 m/z	- 20.0 eV	3.30 min
d_9_-Caffeine (Internal Standard)	204.30 m/z ➔ 144.10 m/z	- 20.0 eV	3.30 min

Data acquisition and analysis were done by using LabSolutions (version 5.97 SP1). Calibration curves consisted of 8 calibration standards and were constructed by plotting peak area ratios of the analytes and internal standards against the concentration of the analytes. A 1/c^2^ weighted least squares linear regression was used for all analytes. The quantitation range was 10–2000 ng/mL.

The LC-MS/MS method was validated in accordance with the Guideline on Bioanalytical Method Validation of the EMA, regarding within- and between-run accuracy and precision, freeze and thaw stability (3 cycles), short term stability at room temperature (24 h) and autosampler stability (48 h) for all analytes in parallel (European Medicines Agency, C. for M.P. for H.U, 2019). The validation results met the acceptance criteria of the EMA Guideline (accuracy: ± 15 % (LLOQ ±20 %), precision: RSD < 15 % (LLOQ RSD < 20 %)).

### 2.7 Calculations for gastric content volumes and emptied fractions

ΔGCV was calculated by subtraction of the postprandial volume right before ingestion of the test volume (volume at *t* = −6 min) from the measured volume of every timepoint from *t* = 4 min to *t* = 58 min. For graphic visualisation the ΔGCV at *t* = 0 min was set to the applied volume of water, e.g., 50, 100, 150 mL.

To obtain a correction for the background emptying of chyme and gastric fluid secretion, a subject- and study arm-individual linear regression was performed with the measuring time points t = 0, 40, 50, 58 min and the respective ΔGCV values (eq. 1). These timepoints were used as water emptying was mostly finished at this time. The obtained individual linear equation was used to calculate the gastric water volume (GWV) in the stomach by subtracting the value on the regression curve from ΔGCV at the respective time point (eq. 2). The obtained volumes were then normalized and subtracted from 1 to display the fraction of water emptied from the stomach (eq. 3). The calculation is given in eqs. 1–3, where *V* is volume, *m* is the gradient of the straight line, *t* is time and *n* is the axis intercept.

Linear regression equation (eq. 1):

V=m∗t+n

Gastric water volume (GWV) calculation (eq. 2):

$$V_{\mathrm{GWV}\left(t\right)}=V_{∆\mathrm{GCV}\left(t\right)}-\left(m*t+n\right)$$

Fraction of water emptied from the stomach (eq. 3):

$$1-\frac{V_{\mathrm{GWV}\left(t\right)}}{V_{\mathrm{GWV}\left(\max\right)}}$$

Saliva concentrations of the two different tracer substances, regular caffeine and ^13^C_3_-caffeine, were baseline corrected using the values obtained at *t* = −5 min in case they were above the limit of quantification (10 ng/ml) and normalized relative to c_max_ afterwards in order to compare them to the fraction of water emptied from the stomach as already described previously (Tzakri et al., 2023; Sager et al., 2018).

GraphPad Prism 5 (version 5.01, Boston, USA) was used to calculate the AUVC_0–58 min_ (area under the volume curve in % ⋅ min) from the fraction of water emptied from the stomach.

### 2.8 Handling of outliers

Possible outliers in the (^13^C_3_-)caffeine-saliva kinetics were detected if individual data points were not supported by two upstream or downstream data points in the trend. These points were identified and discussed with 4 reviewers until consensus on treatment was reached. If the data point was excluded, the mean of the preceding and subsequent data points was calculated and used for further calculations and visualisation. The raw data plots, including labelled excluded data points, can be found in Figs. S 1–9 in the supplementary files.

### 2.9 Statistics

Statistical comparison was performed between the AUVC_0–58 min_ of the gastric water emptying using the fraction of water emptied from the stomach (% ⋅ min). After conformation that the data is normally distributed using Kolmogorov-Smirnov- and Shapiro-Wilk-tests, a paired ANOVA with Tukey multiple comparison test was performed, assuming a significance niveau of α = 0.05. For comparison of the salivary (^13^C_3_-)caffeine appearance from the dosage forms, AUCs_0–59 min_ (ng/mL ⋅ min) from tablet and capsule were tested with a paired ANOVA with Tukey multiple comparison test for normally distributed data as indicated by Kolmogorov-Smirnov- and Shapiro-Wilk-tests. Comparison of emptying of water determined by MRI and salivary appearance of marker substances was performed using AUVC_0–58 min_ of water and normalized AUCs_0–59 min_ from the salivary (^13^C_3_)-caffeine kinetics. Due to normally distributed data, the 50 mL study arm had to be compared with a paired ANOVA with Tukey multiple comparison test, For the 100 and 150 mL study arms a Friedman test with Dunn's post-hoc analysis for non-parametric data had to be used. The emptying rates of the meal have been calculated with linear regression as previously described and statistically compared with a paired ANOVA and Tukey multiple comparison test. All statistical calculations were done with GraphPad Prism 5.

## 3 Results

All subjects but one were able to comply with the study protocol. One subject was unable to hold its breath as required for the MR measurement. This subject was listed as a dropout and a new subject was recruited for a total of 12 subjects. No adverse events were observed.

### 3.1 Gastric emptying as observed by MRI

**Fig. 1:**
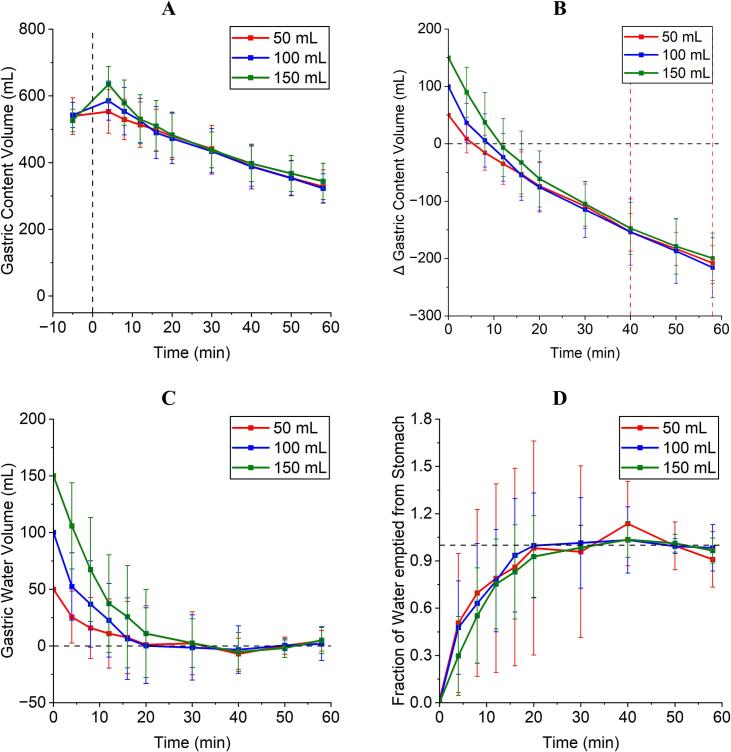
A: Absolute gastric content volume of the three study arms. Dashed line indicates the time point of water ingestion. B: ΔGCV of the three study arms. The dashed black line is indicating a gastric content volume of 0 mL and the dashed red lines indicate the time span used for linear regression to obtain background chyme emptying gastric fluid secretion. C: ΔGWV of the three study arms. The black dashed line is indicating a GWV of 0 mL. D: Fraction of water emptied from the stomach. *N* = 12, mean ± SD. (For interpretation of the references to colour in this figure legend, the reader is referred to the web version of this article.)

As shown in Fig. 1 gastric emptying of the different water volumes in postprandial state was well comparable in the three study arms. Except from the absolute gastric content volume at *t* = 0 min, which is dependent on the administered volume of water, a continuous reduction in gastric contents (Fig. 1A) was observed (individual data in Figs. S 1–9 in the supplementary files). ΔGCV started to become negative as the emptying of chyme from the stomach became the main driver (Fig. 1B). Gastric water volumes (GWV) calculated after subtraction of chyme emptying and gastric secretion as observed in the three study arms is shown in Fig. 1C and the fraction of water emptied from the stomach is displayed in Fig. 1D. Regardless of the ingested volume (50, 100 and 150 mL), water emptying was mostly finished after 20 min, although a high standard deviation was noticeable. No statistically significant differences between the water emptying in the three study arms was recognizable. The AUVC of individual subjects can be seen in Table 4. Gastric emptying of the test meal was similar across all study arms: 3.62 ± 0.54 mL/min (50 mL), 3.73 ± 0.99 mL/min (100 mL), and 3.50 ± 0.83 mL/min (150 mL), with no significant differences observed.

**Table 4:** AUVC_0–58 min_ in % · min of the fraction of water emptied from the stomach of individual subjects.

	AUVC_0–58 min_ 50 mL	AUVC_0–58 min_ 100 mL	AUVC_0–58 min_ 150 mL
P01	34.64	58.87	51.71
P02	53.46	44.49	45.04
P03	25.79	35.91	45.80
P04	65.29	69.67	52.15
P05	44.34	37.80	39.31
P06	42.43	57.40	60.02
P07	31.42	37.00	38.88
P08	64.76	55.16	45.22
P09	57.06	49.05	45.63
P10	50.54	59.77	48.52
P11	57.26	60.66	63.60
P12	95.40	53.34	56.66
**Mean**	**51.87**	**51.59**	**49.39**

### 3.2 Gastric emptying of caffeine from different dosage forms

**Fig. 2:**
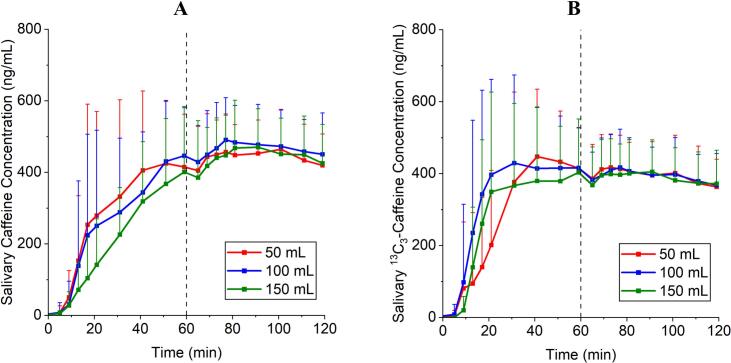
A: Baseline corrected salivary caffeine concentration from the compression-coated tablet in the three study arms. B: Baseline corrected salivary ^13^C_3_-caffeine concentration from the capsule in the three study arms. *N* = 12, mean + SD.

The appearance of the salivary tracers from the two dosage forms differed from the emptying of water from the stomach and was slower on average, especially for the tablet, regardless of the study arm. After drinking 240 mL of water at time t = 60 min, an increase in the saliva concentration was recognizable for both dosage forms in the mean data and in most individual subjects (Fig. 2 and supplementary files). A statistical comparison for the AUC_0–59 min_ of the raw data (ng/mL ⋅ min) with a paired ANOVA and Tukey's multiple comparison test revealed no difference between the three study arms for tablet and capsule. The AUC of individual subjects can be seen in Table 5. The ^13^C_3_-caffeine concentrations in saliva derived from the capsules showed a steeper increase in comparison to the regular caffeine from the tablets.

**Table 5:** AUCs_0–59 min_ in ng/mL · min of saliva concentrations of the different markers caffein and ^13^C_3_-caffeine from tablet and capsule of individual subjects.

	AUC_0–59 min_ Tablet 50 mL	AUC_0–59 min_ Tablet 100 mL	AUC_0–59 min_ Tablet 150 mL	AUC_0–59 min_ Capsule 50 mL	AUC_0–59 min_ Capsule 100 mL	AUC_0–59 min_ Capsule 150 mL
P01	19893	9169	0	10847	5677	4473
P02	38990	22996	15862	27026	35893	28234
P03	9924	11396	18243	15245	13890	25899
P04	28662	25214	15568	23542	19821	12082
P05	2642	7798	8328	21175	16601	27525
P06	13077	10364	6534	16699	22343	17597
P07	20623	9479	13562	10335	5626	1553
P08	16959	15549	16465	15866	22308	19186
P09	2086	5824	3569	5006	16597	8196
P10	14342	36224	15532	26963	34506	15006
P11	15251	14179	13083	18141	16832	18010
P12	17829	16153	19713	7183	22341	21729
**Mean**	**16690**	**15362**	**12205**	**16502**	**19370**	**16624**

### 3.3 Intragastric localisation of the dosage forms in the MR images

**Fig. 3:**
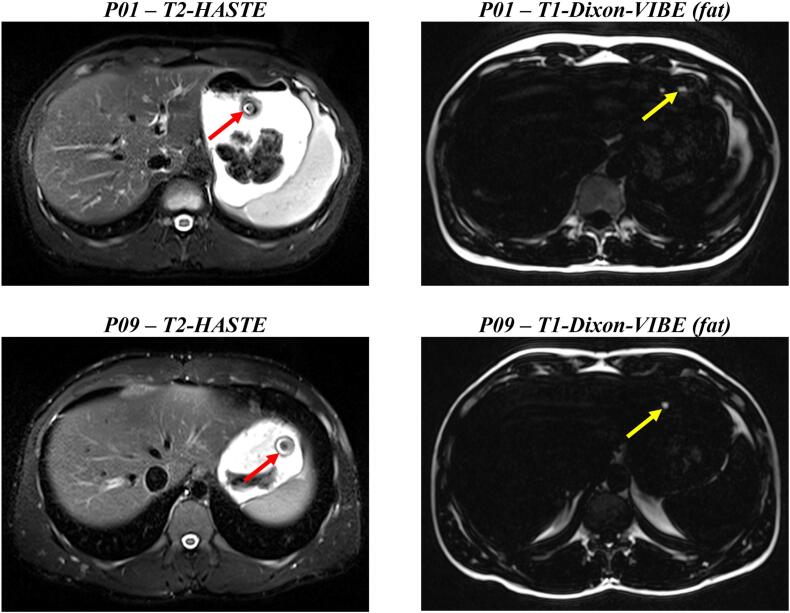
Example images for the localisation of the iron oxide labelled tablet in the HASTE-sequences and the MCT-labelled capsule in the Dixon-VIBE-sequences (fat images). The red (tablet) and yellow (capsule) arrow indicate the location at time point *t* = 4 min for P01 and P09. (For interpretation of the references to colour in this figure legend, the reader is referred to the web version of this article.)

The visibility of the dosage forms in the MR images was not always given. If the tablet was visible, it was mostly located in contact with or surrounded by chyme, identifiable by a typically ring-shaped susceptibility artefact. Tracking of the capsule was more difficult, since sometimes no fat signal was recognizable in the images obtained using the Dixon VIBE sequence. If a signal was visible, it was always located as a small signal-intense spot floating on top of the stomach fillings. In Fig. 3, example images of the different sequences and locations of the dosage forms are shown. Tables S 1–3 in the supplementary files display if and for how long tablets and capsules were visible.

## 4 Discussion

Using a three-armed, randomised, crossover MRI study in 12 subjects in combination with saliva tracers, we evaluated whether the *Magenstrasse* is also present for consumed water volumes below the 240 mL recommended by the FDA and thus regularly used in bioavailability and bioequivalence investigations. For this purpose, 50, 100 and 150 mL of water together with two different immediate release dosage forms, a compression-coated tablet and a hard gelatine capsule, were taken 30 min after consumption of a light meal. Except for the butter, this more representative meal has previously been used in studies showing similar postprandial gastric emptying of water compared to the FDA standard meal for bioequivalence testing (Grimm et al., 2017; Sager et al., 2019a). In the first 58 min after ingestion, MRI measurements were carried out and the emptying of gastric content was determined from the obtained images using computer-aided volumetry. The tablet contained regular caffeine, and the gelatine hard capsule contained stable isotope-labelled ^13^C_3_-caffeine, allowing in vivo release behaviour to be distinguished between the two dosage forms based on the appearance of the two caffeine variants in saliva samples each collected 1 min after MR imaging. Furthermore, the intragastric localization of the capsule and the tablet was tracked using MRI. The emptying of water from the stomach could be successfully determined as the ΔGCV using the method described by Grimm et al. (Grimm et al., 2017). In addition, by subtracting the background gastric emptying of chyme, which was in the range of 3–4 mL/min, and equilibrium secretion of gastric fluids, it was also possible to calculate the theoretical gastric water volume (GWV). The data show, that independent of the different consumed volumes, gastric water emptying was mostly finished within 20 min. This suggests higher flow rates for greater amounts of applied water and lower flow rates for small amounts of water, in line with the first order kinetics reported for gastric water emptying (Tzakri et al., 2023; Kiyota et al., 2022b; Feher, 2012). Gastric water emptying constants were calculated on an individual subject basis with a simple linear fit on natural logarithms of water volumes, although most individual regression coefficients did not allow for robust estimation of exponential emptying. However, regression coefficients increased with the volume of water administered. Estimated water emptying constants were approximately 0.10 min^−1^, 0.15 min^−1^, and 0.13 min^−1^ for the 50, 100, and 150 mL study groups, respectively. This data should be handled with care but is in the range of data from other studies using up to 240 mL under fasted or fed conditions (Grimm et al., 2017; Koziolek et al., 2014; Tzakri et al., 2023; Sager et al., 2018; Feher, 2012; Mudie et al., 2014; Tzakri et al., 2024).

**Fig. 4:**
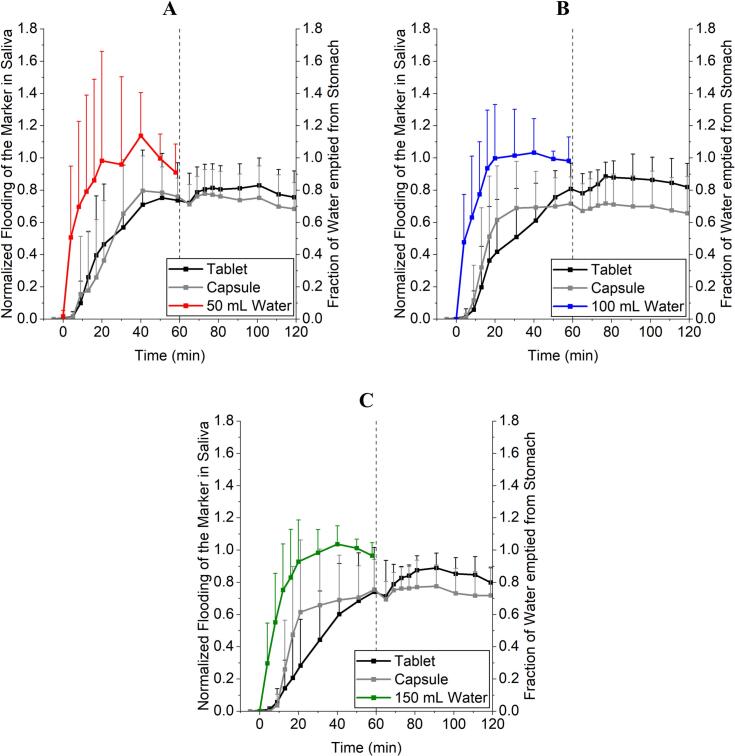
Comparison of the gastric emptying of water observed with MRI, caffeine from a compression-coated tablet and ^13^C_3_-caffeine from a hard-gelatine capsule using saliva samples. A: 50 mL. B: 100 mL. C: 150 mL. *N* = 12, mean + SD.

The compression-coated caffeine tablet was used to label simultaneously applied water after its fast disintegration and to examine gastric emptying of a hydrophilic and highly permeable model substance (Tzakri et al., 2023; Sager et al., 2018; Sager et al., 2019b). In this setting, both tablet and capsule seem to miss the onset and first minutes of water emptying as shown by MRI, Fig. 4 and Table 6. This could be for both dosage forms due to different reasons which contribute to a certain lag-time in release. Despite a positioning of the capsule mostly surrounded by water, the disintegration time was too slow, so that some of the co-administered water was emptied faster than the disintegration progressed. In case of the tablet: It was mostly positioned surrounded by chyme and regardless of a possible fast disintegration, caffeine was not able to be suspended and/or dissolved in the water and to be carried along by the water phase which was evacuated fast from the stomach. The fraction of water emptied from the stomach was corrected for background emptying of chyme and secretion of gastric fluids, whereas the normalized flooding of the marker in saliva was not corrected for simultaneously proceeding elimination processes. Although the elimination half-life (t_1/2_) of caffeine is described as fast with around 4 h, especially the onset and the overall short observation period preserves the general informative value of the profiles (Alsabri et al., 2018). Elimination-corrected values of caffeine would most likely result in slightly higher AUCs but not in extensively changed graph geometry.

**Table 6:** Statistical comparison of (^13^C_3_-) caffeine appearance in saliva (normalized AUC_0–59 min_ in %·min) and fraction of water emptied from the stomach (AUVC_0–58 min_ in %·min) using paired ANOVA with Tukey's multiple comparison test for normally distributed data or Friedman test and Dunn's multiple comparison test for non-parametric data. *N* = 12.

50 mL	
	**Significance/*P*-value (Tukey)**
Water vs. Caffeine (Tablet)	< 0.01
Water vs. ^13^C_3_-Caffeine (Capsule)	< 0.01
Caffeine (Tablet) vs. ^13^C_3_-Caffeine (Capsule)	non significant
**100 mL**	
	**Significance/P-value (Dunn)**
Water vs. Caffeine (Tablet)	< 0.001
Water vs. ^13^C_3_-Caffeine (Capsule)	< 0.01
Caffeine (Tablet) vs. ^13^C_3_-Caffeine (Capsule)	non significant
**150 mL**	
	**Significance/P-value (Dunn)**
Water vs. Caffeine (Tablet)	< 0.001
Water vs. ^13^C_3_-Caffeine (Capsule)	< 0.05
Caffeine (Tablet) vs. ^13^C_3_-Caffeine (Capsule)	non significant

Additional drinking of 240 mL water, 60 min after intake of the dosage form led to a visible small second peak in the saliva concentration of the different caffeine molecules, indicating incomplete dissolution in the stomach and sedimentation or mixing with the mostly hydrophilic light meal. When drinking another 240 mL water after some time, new dissolution medium, a high concentration gradient and a high flow rate could have dissolved and carried substances left behind in the stomach after the first *Magenstrasse.* In bioequivalence studies, water ad libitum is allowed beginning from one hour after dosing, which in this case could lead to inexplicable plasma peaks. For interpretation of these clinical data, it appears to be necessary to stick to a strict study protocol with set amounts and intervals of water consumption or at least a documentation of volumes and time points should be considered (Grimm et al., 2017).

The used capsule had a low density, resulting in a floating dosage form. If the capsule was visible in the MR images, it always floated on top of the chyme or watery phase. When surrounded by chyme MRI visibility was not achieved as the food also partially shows positive contrast in T1-weighted imaging. The supine posture could have resulted in more contact time with the dissolution medium for the capsule, speeding up the gastric emptying of the ^13^C_3_-caffeine. In this case, the drug substance in the capsule could have had more contact time with the *Magenstrasse*, which led to a slightly faster gastric emptying in comparison to the tablet which nevertheless was statistically non-significant (Fig. 4 and Table 6). The tablet, which has a higher density, was always surrounded or embedded in chyme, sometimes laying right below the lower esophageal sphincter in the *cardia*-region of the stomach. The T1-VIBE-images, which have originally been used to locate the capsules, sometimes revealed signs of suspended iron oxide particles in the chyme (small susceptibility artefacts), when the typical large susceptibility artefact of the tablets core wasn't visible anymore in the HASTE-sequences (Bekiesińska-Figatowska, 2015). This could be one reason for a limited contact time with the simultaneously applied water and a resulting slower appearance in the saliva. Since the dosage forms were ingested in an upright position and directly after, the subjects laid on their back inside the MR scanner for one hour, the posture dependent positioning of the dosage forms in the stomach may have led to the differences between gastric water emptying and emptying of the salivary tracers. These observations and the gastric water emptying could therefore be different when measuring in an upright position. Tzakri et al. showed in their studies that the so called “ice capsule”, a dosage form designed to label water evacuating from the stomach with caffeine, and the fast disintegrating, compression-coated tablet to evaluate gastric water emptying were statistically interchangeable even under postprandial conditions (Tzakri et al., 2023; Sager et al., 2018). In contrast to this study, volunteers were positioned upright during the whole time and got a standardised high-calorie, high-fat meal as recommended by the FDA guidance. Especially the differences between the light meal (mostly hydrophilic) and the high-calorie meal (more lipophilic) could have influenced the distribution ratio on the hydrophilic marker caffeine mixing with the respective chyme (Winter et al., 2023).

In the study protocol used here, water emptying and transfer of a drug released from a solid oral dosage form to the duodenum cannot be set equal. Since this study was conducted with a healthy and young population, the results cannot be transferred directly to a diseased population. It is known that various diseases such as diabetes mellitus, gastritis, reflux or neurological issues have an influence on gastric emptying (Choung et al., 2012; Tatsuta et al., 1989; Emerenziani and Sifrim, 2005; Fasano et al., 2015).

Individual profiles (Figs. S 1–9) in comparison to the visibility of the dosage forms in the MR images (Tables S 1–3) revealed that caffeine often appeared a little earlier in saliva than the disintegration of the dosage form was noticeable. For the capsule this could be due to the fact that the MCT was encapsuled in a separate smaller capsule, which had to dissolve after disintegration of the outer capsule. For the tablet however, the non-soluble marker iron oxide could have sedimented to form a “look-alike” of the tablet core. If the concentrated susceptibility artefact disappeared, the dispersed iron oxide was often visible in the corresponding T1-VIBE-sequences as mentioned above. As the fundus is not responsible for mixing and grinding of the chyme, it is unlikely that sedimented material of the dosage form quickly changes its localisation if the body position is not altered (Hennig and Spencer, 2018; Goyal et al., 2019). For example in subject P01, the tablet wasn't visible at all in the 50 and 100 mL study arms, which matched the saliva profiles, as caffeine appeared rapidly in the early saliva samples. In this case the tablet could have disintegrated fast in the stomach and took the first emptying of water or the tablet was directly emptied into the duodenum and disintegrated in the small intestine. In contrast, in the 150 mL study arm, the susceptibility artefact was visible for 40 min, whereas the saliva concentration started to rise not earlier than after 60 min, indicating that the tablet may have disintegrated with the first applied volume of water but the sediments layed trapped in or under the chyme so that the caffeine could not take the path of the first applied water. Sometimes the visibility of the dosage form did not match the saliva appearance (e.g. P04, capsule, 150 mL) which could have occurred due to low mixing of the already disintegrated dosage form, or the reasons mentioned above.

In contrast to caffeine, most active pharmaceutical ingredients (API) are poorly soluble (BCS class II or IV) (Ting et al., 2018; Kawabata et al., 2011; Bhalani et al., 2022). The data from this study should therefore not be misinterpreted to mean that it does not matter whether patients should take a lot or a little liquid with their medication. For water and real solutions, it could be assumed that a fast emptying from the stomach in fasted and fed conditions is likely to happen irrespective of the volume taken. If there is a dosage form which is dependent on some kind of liberation or dissolution it could be possible that due to sedimentation of possibly poorly soluble API or mixing with chyme, not the whole dose gets evacuated fast from the stomach (Van Den Abeele et al., 2017; Van Den Abeele et al., 2016; Koenigsknecht et al., 2017; Paixão et al., 2018; Tomilo et al., 2006; Lee et al., 2022). For these dosage forms, an increased amount of water ingested alongside could be beneficial, not only to avoid incomplete passage through the esophagus and related side effects (Kim, 2014; Dag et al., 2014). Both increased hydrostatic pressure and increased gastric wall tension could contribute to higher absolute flow rates in terms of mL/min (Grimm et al., 2023b). This could possibly influence the transport and entrainment of suspended particles and also increases the available solvent volume. In these cases in particular, drinking enough fluid some time after intake of the dosage form could help to speed up the passage through the stomach, which may explain parts of observed unexpected plasma peaks from study personnel in clinical food effect studies. The renewed appearance in the saliva as seen here after 60 min supports these assumptions.

## 5 Conclusion

Using a combined MRI and saliva study with 12 volunteers and two differently labelled dosage forms we found that gastric water emptying in the postprandial state after consumption of a light meal is independent of the applied volume of water (50, 100, 150 mL) and comparable to data from the literature using 240 mL water. This indicates that the *Magenstrasse* or stomach road also exists and is recognizable using MRI under the application of low liquid volumes of only 50 mL. 60 min after postprandial intake of the dosage forms, 240 mL of water were able to flush out remaining well soluble drug substance from oral dosage forms, irrespective of the volume they have been taken with. However, whether a substance from an oral solid dosage form is entrained by water depends on the type of the dosage form itself, the posture, the intragastric localisation, the speed of dispersion formation and possibly also the combination of the type of meal and the physico-chemical properties of the applied active substance.

## Clinical trial registration

The study was registered at the German Register for Clinical trials under DRKS00033695.

## Subject consent

All study participants gave written informed consent on study conduction, including data handling and publication.

## CRediT authorship contribution statement

**Linus Großmann:** Writing – original draft, Visualization, Supervision, Methodology, Investigation, Formal analysis, Conceptualization. **Johanna Cyrus:** Investigation, Formal analysis. **Stefan Senekowitsch:** Validation, Formal analysis. **Toni Wildgrube:** Validation, Formal analysis. **Theodora Tzakri:** Methodology, Formal analysis. **Marie-Luise Kromrey:** Project administration. **Werner Weitschies:** Writing – review & editing, Supervision, Project administration, Conceptualization. **Michael Grimm:** Writing – review & editing, Supervision, Project administration, Methodology, Formal analysis.

## Ethics approval

The study was approved by the Ethics Committee of the University Hospital of Greifswald under registration number BB 080/23.

## Funding

The University of Greifswald received funding from the German Research Council (DFG INST 292/155-1 FUGG).

## Declaration of competing interest

The authors declare no conflicts of interest.

## Data Availability

Raw data will be made available upon reasonable request.

## Appendix A Supplementary data

Supplementary material Image 1
