# Supplementary material for: Does the appearance of the Magenstrasse depend on the amount of water consumed?
Source: Int J Pharm X. 2025 Jul 23;10:100365. doi: 10.1016/j.ijpx.2025.100365 (PMC12320173; doi:10.1016/j.ijpx.2025.100365)
Supplement: Supplementary file 1 — Supplementary material [file mmc1.pdf]

Supplementary files - Does the appearance of the Magenstrasse depend on the amount of water consumed?

**P001**	time (min)	4	8	12	16	20	30	40	50	60	visible to (min)
50 mL	tablet	-	-	-	-	-	-	-	-	-	0
	capsule	+	+	+	+	+	+	-	-	-	30
100 mL	tablet	-	-	-	-	-	-	-	-	-	0
	capsule	-	+	+	+	+	+	-	-	-	30
150 mL	tablet	+	+	+	+	+	+	+	+	-	50
	capsule	+	+	+	+	+	-	-	-	-	20
**P102**	time (min)	4	8	12	16	20	30	40	50	60	visible to (min)
50 mL	tablet	+	+	-	-	-	-	-	-	-	8
	capsule	+	+	+	-	-	-	-	-	-	12
100 mL	tablet	-	-	-	-	-	-	-	-	-	0
	capsule	+	-	-	-	-	-	-	-	-	4
150 mL	tablet	+	+	+	-	-	-	-	-	-	12
	capsul	+	+	-	-	-	-	-	-	-	8
**P003**	time (min)	4	8	12	16	20	30	40	50	60	visible to (min)
50 mL	tablet	+	+	+	+	+	+	-	-	-	30
	capsule	+	+	+	-	-	-	-	-	-	12
100 mL	tablet	+	+	+	+	+	-	-	-	-	20
	capsule	+	+	+	+	+	+	-	-	-	30
150 mL	tablet	+	+	+	+	-	-	-	-	-	16
	capsule	+	-	-	-	-	-	-	-	-	4
**P004**	time (min)	4	8	12	16	20	30	40	50	60	visible to (min)
50 mL	tablet	-	-	-	-	-	-	-	-	-	0
	capsule	-	-	-	-	-	-	-	-	-	0
100 mL	tablet	-	-	-	-	-	-	-	-	-	0
	capsule	+	+	-	-	-	-	-	-	-	8
150 mL	tablet	-	-	-	-	-	-	-	-	-	0
	capsule	+	+	+	+	+	+	-	-	-	30

Table S 1 Visibility of dosage forms in MRI. + = visible, - = not visible. P001-P004.

**P005**	time (min)	4	8	12	16	20	30	40	50	60	visible to (min)
50 mL	tablet	+	+	+	+	+	+	+	-	-	40
	capsule	+	+	+	+	-	-	-	-	-	16
100 mL	tablet	+	+	+	+	+	+	+	+	-	50
	capsule	+	+	-	-	-	-	-	-	-	8
150 mL	tablet	+	+	+	+	+	-	-	-	-	20
	capsule	-	-	-	-	-	-	-	-	-	0
**P006**	time (min)	4	8	12	16	20	30	40	50	60	visible to (min)
50 mL	tablet	+	+	-	-	-	-	-	-	-	8
	capsule	+	+	+	-	-	-	-	-	-	12
100 mL	tablet	+	-	-	-	-	-	-	-	-	4
	capsule	-	-	-	-	-	-	-	-	-	0
150 mL	tablet	+	+	+	+	+	+	-	-	-	30
	capsule	-	-	-	-	-	-	-	-	-	0
**P007**	time (min)	4	8	12	16	20	30	40	50	60	visible to (min)
50 mL	tablet	+	+	-	-	-	-	-	-	-	8
	capsule	+	+	+	+	-	-	-	-	-	16
100 mL	tablet	+	+	+	+	+	+	-	-	-	30
	capsule	-	-	+	+	+	+	+	-	-	40
150 mL	tablet	-	-	-	-	-	-	-	-	-	0
	capsule	+	+	+	+	+	+	+	-	-	40
**P008**	time (min)	4	8	12	16	20	30	40	50	60	visible to (min)
50 mL	Tablette	-	-	-	-	-	-	-	-	-	0
	Kapsel	+	-	-	-	-	-	-	-	-	4
100 mL	Tablette	-	-	-	-	-	-	-	-	-	0
	Kapsel	-	-	-	-	-	-	-	-	-	0
150 mL	Tablette	-	-	-	-	-	-	-	-	-	0
	Kapsel	-	+	+	-	-	-	-	-	-	12

Table S 2 Visibility of dosage forms in MRI. + = visible, - = not visible. P005-P008.

**P009**	time (min)	4	8	12	16	20	30	40	50	60	visible to (min)
50 mL	tablet	+	+	+	+	+	+	+	-	-	40
	capsule	+	+	+	+	+	+	+	-	-	40
100 mL	tablet	+	+	+	+	+	-	-	-	-	20
	capsule	-	-	+	-	-	-	-	-	-	12
150 mL	tablet	+	+	+	+	+	-	-	-	-	20
	capsule	+	+	+	+	+	-	-	-	-	20
**P010**	time (min)	4	8	12	16	20	30	40	50	60	visible to (min)
50 mL	tablet	+	+	+	-	-	-	-	-	-	12
	capsule	+	-	-	-	-	-	-	-	-	4
100 mL	tablet	+	+	-	-	-	-	-	-	-	8
	capsule	-	-	-	-	-	-	-	-	-	0
150 mL	tablet	-	-	-	-	-	-	-	-	-	0
	capsule	+	+	+	+	+	+	+	-	-	40
**P011**	time (min)	4	8	12	16	20	30	40	50	60	visible to (min)
50 mL	tablet	-	-	-	-	-	-	-	-	-	0
	capsule	-	-	-	-	-	-	-	-	-	0
100 mL	tablet	+	+	-	-	-	-	-	-	-	8
	capsule	+	+	-	-	-	-	-	-	-	8
150 mL	tablet	+	+	-	-	-	-	-	-	-	8
	capsule	+	+	-	-	-	-	-	-	-	8
**P012**	time (min)	4	8	12	16	20	30	40	50	60	visible to (min)
50 mL	tablet	+	+	+	-	-	-	-	-	-	12
	capsule	+	+	+	+	+	-	-	-	-	20
100 mL	tablet	+	+	-	-	-	-	-	-	-	8
	capsule	-	-	-	-	-	-	-	-	-	0
150 mL	tablet	-	-	-	-	-	-	-	-	-	0
	capsule	-	-	-	-	-	-	-	-	-	0

Table S 3 Visibility of dosage forms in MRI. + = visible, - = not visible. P009-P010.

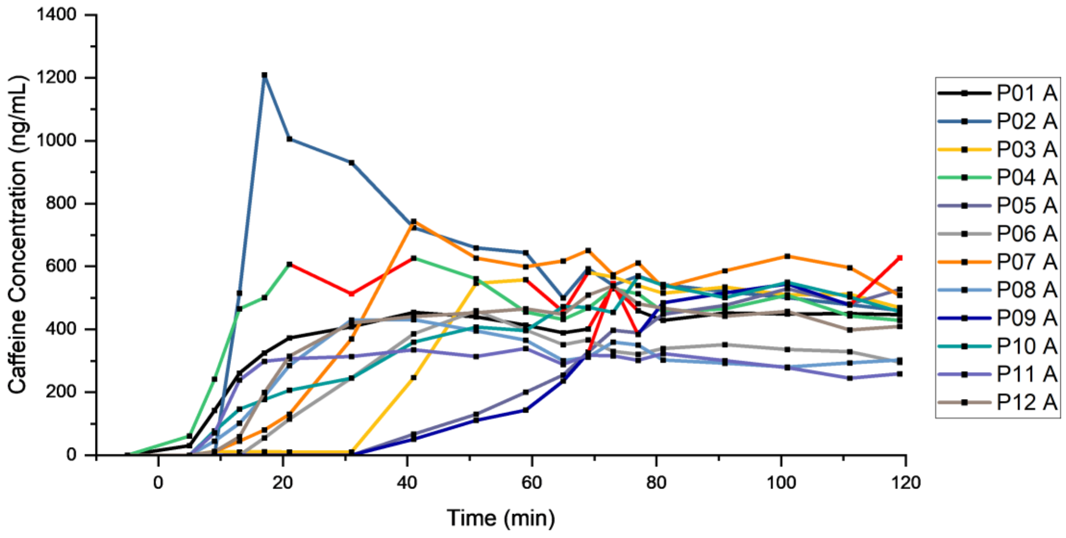

Figure S 1 Individual baseline corrected data for caffeine concentration in saliva. Tablet taken with 50 mL water. Red areas and points are labelled outliers.

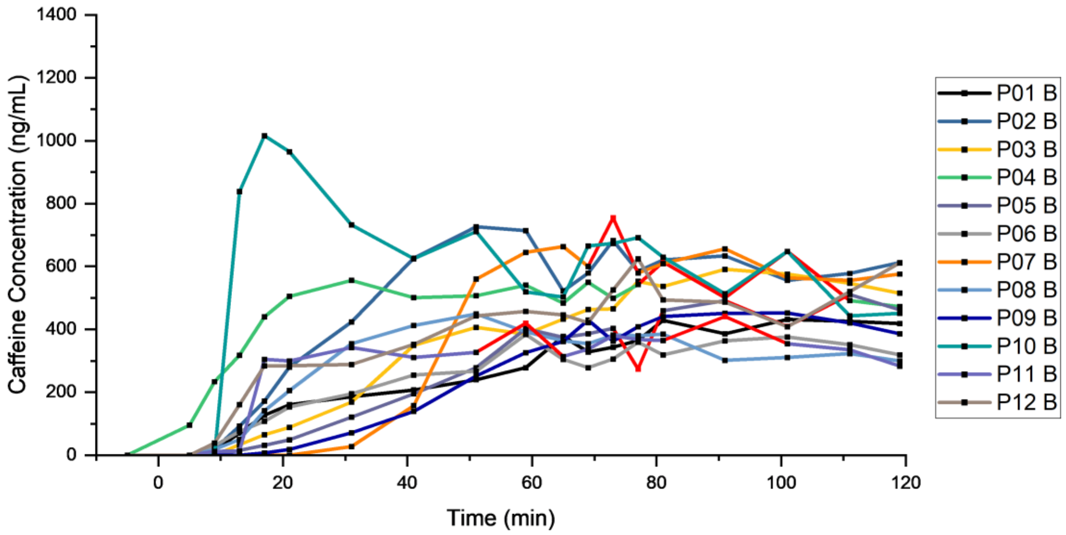

Figure S 2 Individual baseline corrected data for caffeine concentration in saliva. Tablet taken with 100 mL water. Red areas and points are labelled outliers.

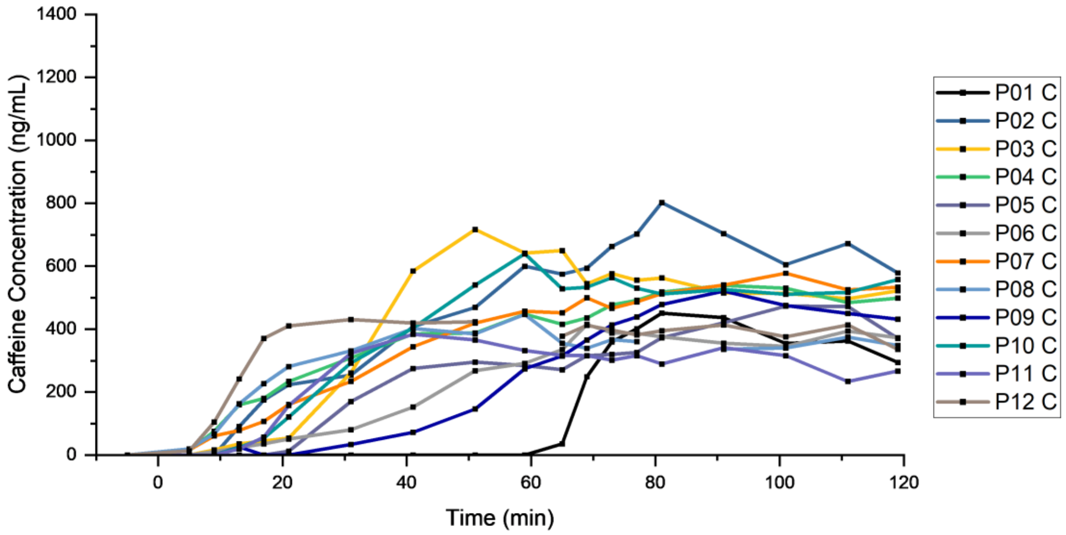

Figure S 3 Individual baseline corrected data for caffeine concentration in saliva. Tablet taken with 150 mL water.

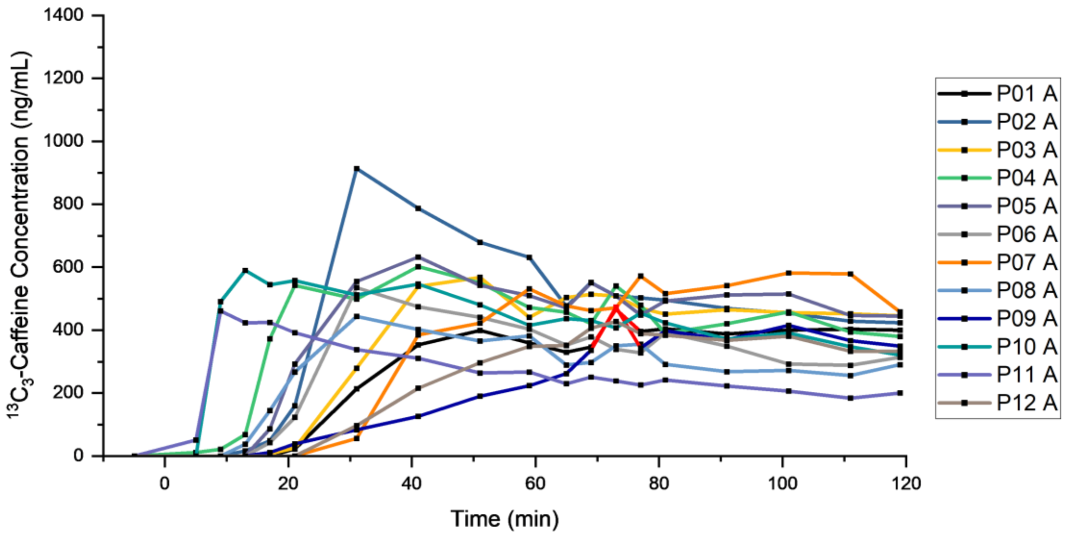

Figure S 4 Individual baseline corrected data for $^{13}C_3$-caffeine concentration in saliva. Capsule taken with 50 mL water. Red areas and points are labelled outliers.

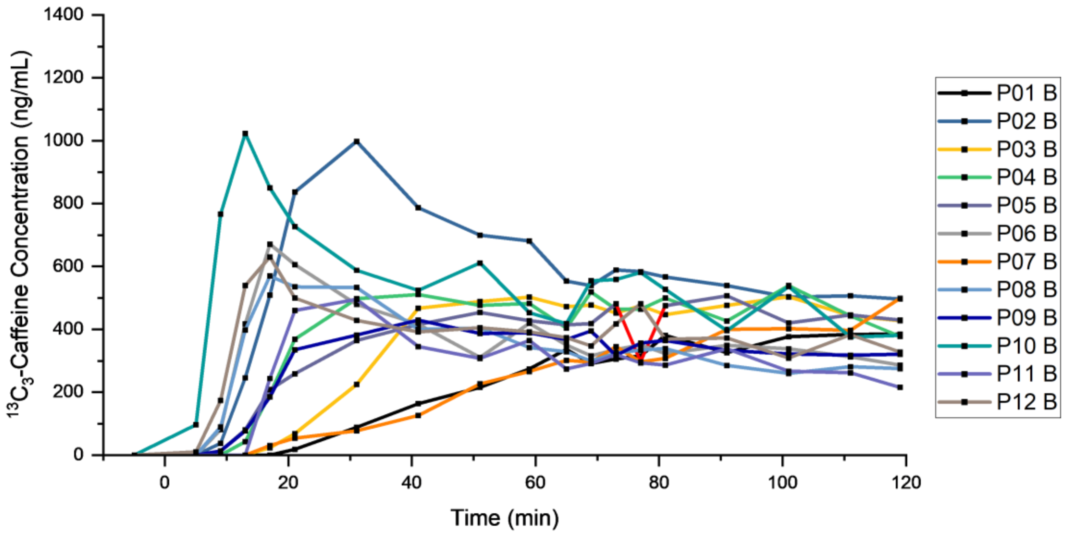

Figure S 5 Individual baseline corrected data for $^{13}C_3$-caffeine concentration in saliva. Capsule taken with 100 mL water. Red areas and points are labelled outliers.

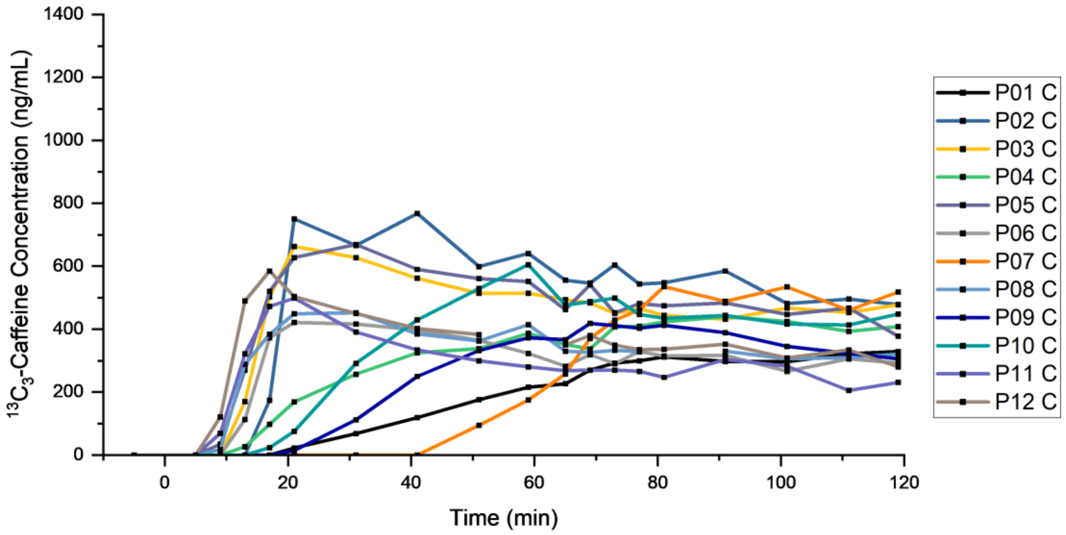

Figure S 6 Individual baseline corrected data for $^{13}C_3$-caffeine concentration in saliva. Capsule taken with 150 mL water.

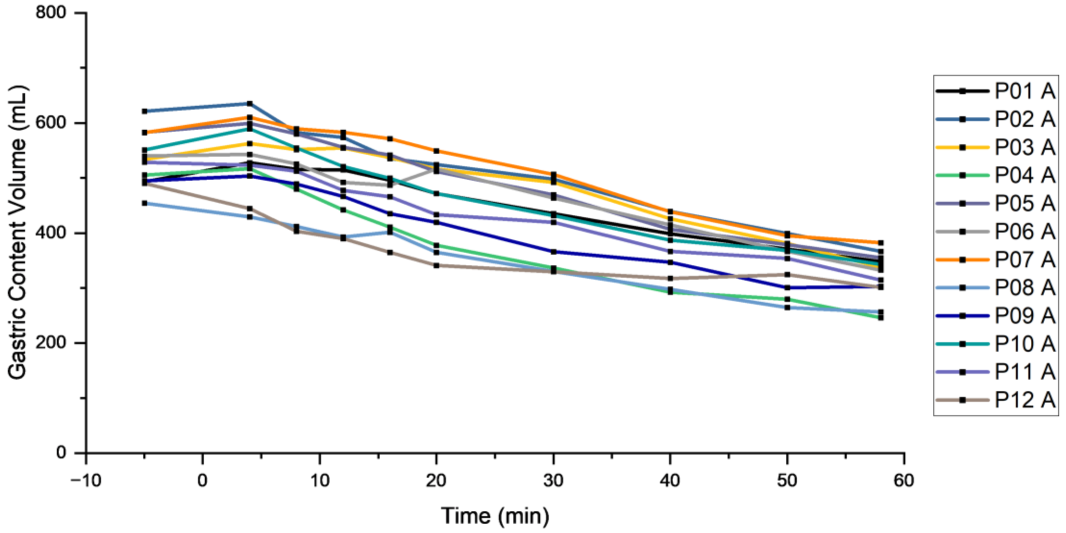

Figure S 7 Individual gastric content volume after ingestion of a light meal and 50 mL water.

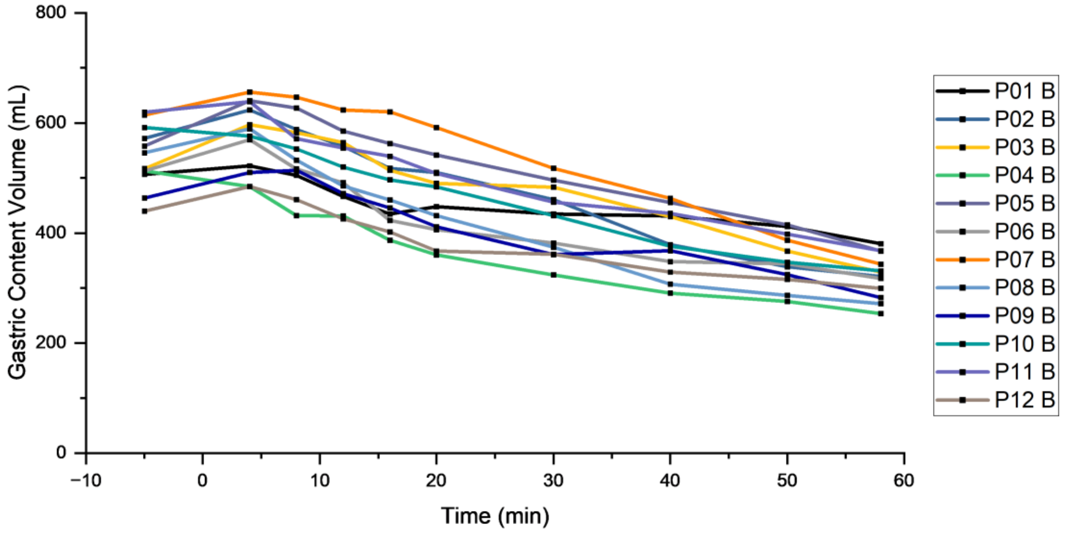

Figure S 8 Individual gastric content volume after ingestion of a light meal and 100 mL water.

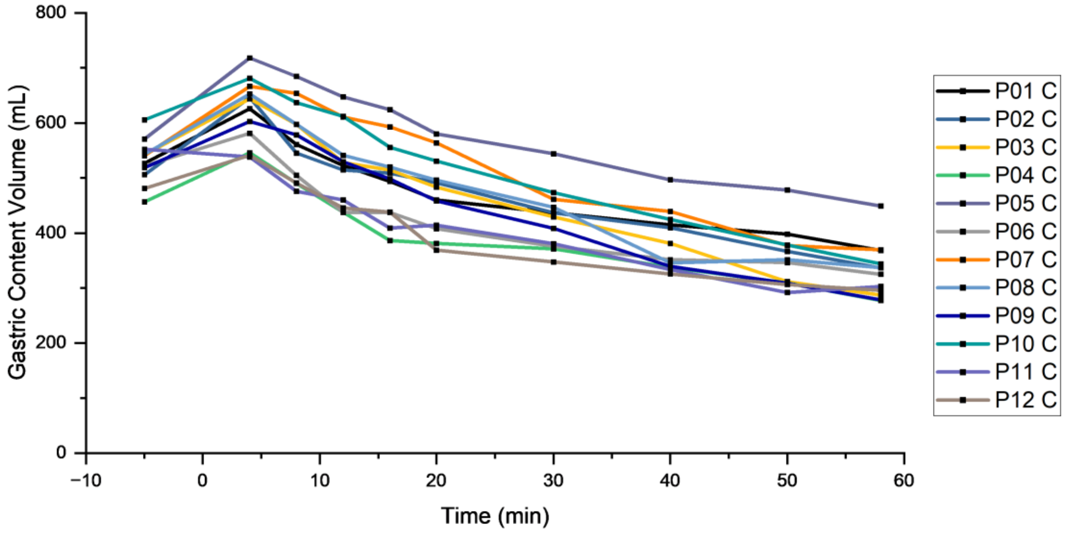

Figure S 9 Individual gastric content volume after ingestion of a light meal and 150 mL water.
